# Dietary intake of fibers: differential effects in men and women on perceived general health and immune functioning

**DOI:** 10.1080/16546628.2017.1297053

**Published:** 2017-03-23

**Authors:** Amanda M. Fernstrand, Didi Bury, Johan Garssen, Joris C. Verster

**Affiliations:** ^a^ Division of Pharmacology, Utrecht University, Utrecht, the Netherlands; ^b^ Nutricia Research, Utrecht, the Netherlands; ^c^ Centre for Human Psychopharmacology, Swinburne University, Melbourne, Australia; ^d^ Institute for Risk Assessment Sciences, Utrecht University, Utrecht, the Netherlands

**Keywords:** Fiber, microbiome, dysbiosis, perceived immune functioning, gender, health status

## Abstract

**Background**: It has been reported previously that dietary fiber intake provides health benefits. Nevertheless, only a limited number of human studies have investigated whether gender differences exist in the relationship between fiber intake and perceived health and immune status.

**Objective**: To investigate potential gender differences in the effects of dietary fiber intake on perceived health and immune status of healthy young adults.

**Design**: A survey was conducted among university students in Utrecht, the Netherlands. Data were collected on perceived general health status and perceived immune functioning. Dietary intake of fibers was assessed using a food frequency questionnaire. Perceived general health status and immune functioning were associated with daily intake of fibers using nonparametric (Spearman) correlations. Statistical analyses were conducted for the group as a whole, and for men and women separately.

**Results**: N = 509 subjects completed the survey. Mean (SD) age was 20.8 (2.6) years old. 71.9% of the samples were females. Mean daily dietary fiber intake was 15.5 (6.9) g. Daily dietary fiber intake correlated significantly with general health rate (r = 0.171, *p *= 0.0001) and perceived immune functioning (r = 0.124, *p *= 0.008). After controlling for total caloric intake, the partial correlation between fiber intake and general health remained significant (r = 0.151, *p *= 0.002). In men, dietary fiber intake correlated significantly with perceived general health status (r = 0.320, *p *= 0.0001) and immune functioning (r = 0.281, *p *= 0.002). After controlling for caloric intake, the association between dietary fiber intake and perceived general health (r = 0.261, *p *= 0.005) remained significant. Remarkably, no significant correlations were observed in women.

**Conclusion**: A significant association between daily dietary fiber intake and perceived general health status and immune rate was found in men, but not in women. Future studies should further address the nature and causes of the observed gender differences, including validated biomarkers for immune responsiveness.

**Abbreviations:** FFQ: Food frequency questionnaire; GIT: Gastrointestinal tract; NCDs: Non-communicable diseases; SCFA: Short-chain fatty acid

## Introduction

Over the past decades, changes in socio-economic status, population growth and agriculture have led to altered dietary habits.[1] Also, an increasing number of epidemiological observations have highlighted the dramatic increase in the incidence of inflammatory diseases in the ‘Westernized’ world.[2] Previous research has demonstrated the effect of diets on microbial composition, and an increasing number of studies supports the impact of food on both the gut microbiome and immunological pathways.[1,3]

The gut microbiome consists of trillions of microorganisms that have co-evolved with the host in a symbiotic manner.[4,5] The gut microbiome has long been appreciated for the health benefits it provides to the host, which includes supply of essential nutrients, enhancement of metabolic capacities and protection against pathogens.[1,4,6] Furthermore, the microbiome communicates with the immune system and modulates the development of the gastrointestinal tract.[7,8] Dysbiosis, i.e. changes in the relative presence of various gut microbiota, has been associated with the pathogenesis of intestinal disorders such as inflammatory bowel disease and coeliac disease but also with extra-intestinal disorders such as allergy, asthma, metabolic syndrome, cardiovascular disease and obesity.[8] Hence, reduced presence of beneficial microbiota has been suggested to be the underlying cause, at least in part, to the increasing incidence of inflammatory disease and associated non-communicable diseases (NCDs).[2]

Dietary intake of fibers provides many health benefits, including reduced incidence of cardiovascular diseases, diabetes, colon cancer, obesity and certain gastrointestinal disorders. Intake of dietary fibers also appears to improve immune functioning.[9] These findings are supported by studies in both animals and humans.[3] It has been shown that people who consume adequate amounts of dietary fibers have lower incidences of inflammatory diseases.[3,7] Conversely, a low intake of fiber may have adverse effects on microbial composition that could lead to health complications.[3] Although daily intake of fibers has been recommended for many years, Western diets are still characterized by processed and stored food high in fat and refined sugar, but low in fiber content.[2] The recommended adequate intake of fibers is 25–38 g per day for adults (14 g/1000 kcal/day). Yet, the diet of a substantial number of people in developed countries is insufficient to achieve the recommended daily intake of fibers.[9]

Fibers affect immune functioning particularly via the production of short-chain fatty acids.[10] Short-chain fatty acids are produced in the fermentation process of dietary fibers and exert various beneficial effects, including maintenance of epithelial barrier functions, regulation of proliferation and tumor suppression, reduction in oxidative DNA damage, and regulation of cytokine production. They also exhibit several anti-inflammatory effects and appear to have a role in the regulation of timing of immune responses as well as in the resolution of inflammation.[3,11]

The increasing number of studies supporting the association between dietary intake of fibers, the microbiome, and health and immune parameters opens up new approaches in understanding and treating immune related diseases.[3] However, little is known about possible gender differences. Therefore, the aim of this study is to investigate potential gender differences in the association between dietary fiber intake and perceived general health and immune functioning in healthy young adults.

## Methods

### Participants

This study was conducted among Dutch university students. The study population included healthy young adults aged ≥18 to ≤ 30 years old. Participation in the study was voluntary and anonymous, and no formal ethics approval was required to conduct the study. Subjects were informed that a completed questionnaire was considered to give consent to the data being used for scientific purposes only.

### Measures

#### Demographic information

Gender, age, weight and height were recorded in the demographics.

#### Food frequency questionnaire

In this study, an adapted version of the short semi-quantitative food group questionnaire used in the ‘Million Women Study’ was used.[12] Similar adaptions of this food frequency questionnaire (FFQ) have been used successfully in target populations of young adults.[13,14] The FFQ assesses consumption of meat, fish, vegetables and salads, dairy products, snacks and grain products. The participants were first asked what food products they had been consuming during the past week followed by the estimated amounts of those products. Unlike most conventional FFQs, such as the NHANES,[15] the respondents were asked to write down the estimated number of times a food item was eaten during the past week instead of indicating their consumption in pre-defined categories. For foods such as vegetables, fruits and nuts, quantity was estimated for the food groups eaten in number of tablespoons and similar. The consumption of each food item was proportionally allocated according to the amount of respective food group reported using standard portion sizes (USDA) or quantitative measures when applicable. Daily fiber intake was calculated using data from USDA.[16] Macronutrient intake was calculated using council directives on nutrition labeling of foodstuffs.[17]

Participants with extreme or implausible values for energy intake were excluded to avoid under or over-reporting. Subjects with a total energy intake of <800 or >5000 for men and <600 or >4000 for women were removed from further analysis. The cutoff values were based on values used previously.[18]

#### Health- and immune status

Data on perceived health and immune status was collected using 1-item scores ranging from bad to excellent on a scale from 0 to 10. They were asked whether they considered their immune functioning to be reduced or not (yes/no).

### Statistical analysis

All statistical analysis was conducted using SPSS 23. Descriptive statistics were computed for all parameters. For the group as a whole, and for males and females separately, the associations between dietary fiber intake and perceived health and immune status were computed using two-tailed nonparametric Spearman’s rank correlations. Partial correlations were computed to control for daily caloric intake. Gender differences in dietary intake and health parameters were assessed using two-tailed independent-samples Mann–Whitney U-tests. Statistical significance was set at *p *< 0.05.

## Results

### Characteristics

The study included N = 657 participants, of whom N = 148 had missing responses or under/over-reported energy intake. Data from N = 509 participants were used for the statistical analyses, including N = 366 females (71.9%) and 143 (29.1%) males. Mean (SD) age was 20.8 (2.6) years. Mean BMI was 21.6 (2.3) for women and 22.2 (2.3) for men. The response rate was 77%.

### Food consumption and health indicators

Mean (SD) energy intake per day was 1196 (386) kcal/day for women and 1611 (540) kcal/day for men. Mean daily dietary intake of fibers was 15.5 (6.9) g for the total study population, 14.9 (6.5) g for women and 17.3 (7.7) g for men. When controlling for daily energy intake, the daily dietary intake of fibers was 11.9 g/1000 kcal/day for the total study population, 12.5 g/1000 kcal/day for women, and 10.9 g/1000 kcal/day for men (*p *= 0.001). Most fibers come from cereals and grain products (59.7%), followed by fruit (27.1%) and vegetables (5.8%). The relative contribution of these sources to total fiber intake did not significantly differ between men and women for vegetables. However, men consumed significantly more cereals and grain products (*p *= 0.029), whereas women consumed significantly more fruit (*p *= 0.0001). Mean macronutrient intake, as percentage of total energy intake, was 8.3% protein, 58.0% carbohydrates, and 9.4% fat. Macronutrient intake did not differ significantly between the genders.

The mean (SD) general health score was 7.8 (1.0) for the overall population, 7.7 (1.0) for women and 7.9 (1.2) for men. The mean (SD) rating for immune function was 7.8 (1.3) for the total population, 7.7 (1.0) for women and 8.0 (1.5) for men. Regarding perceived immune status, 28.9% of the overall study population reported reduced immunity. In women, 31.9% reported to experience reduced immunity. This number was significantly higher compared to the male population who reported reduced immunity in 21.3% of the cases (*p *= 0.018).

### Associations between dietary intake and perceived health and immune rate

**Figure 1.**
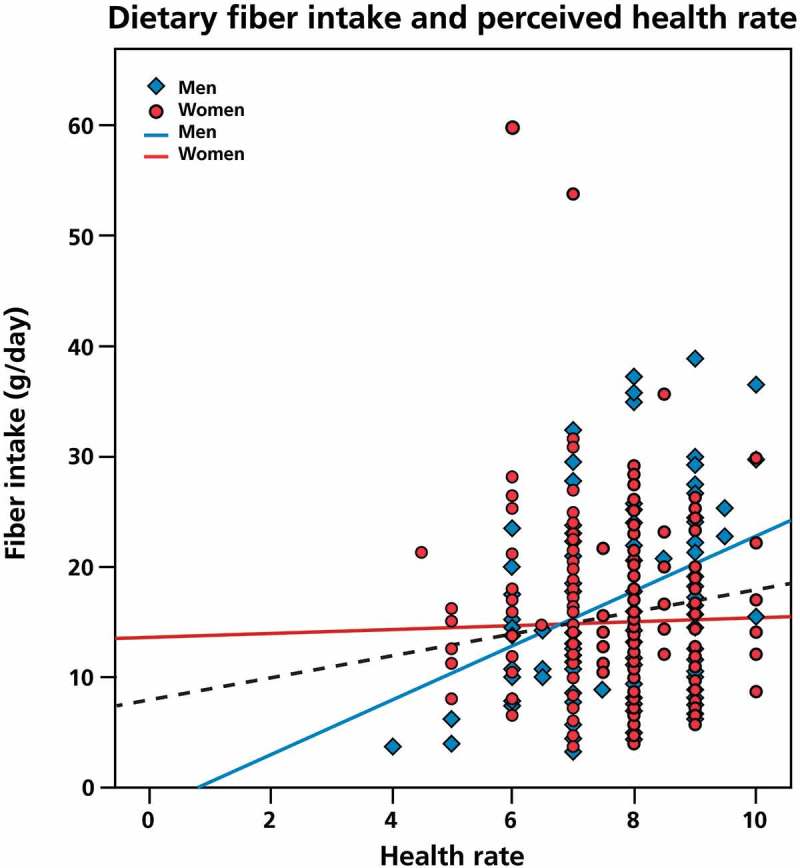
Dietary fiber intake and general health. To explore the possible relationship between self-reported health status and fiber intake, a two-tailed nonparametric Spearman’s correlation was applied. Significant positive correlations were found for the total population (*p *= 0.0001) and for men (*p *= 0.0001). No significant correlation was found between daily intake of fibers and general health in women.

**Figure 2.**
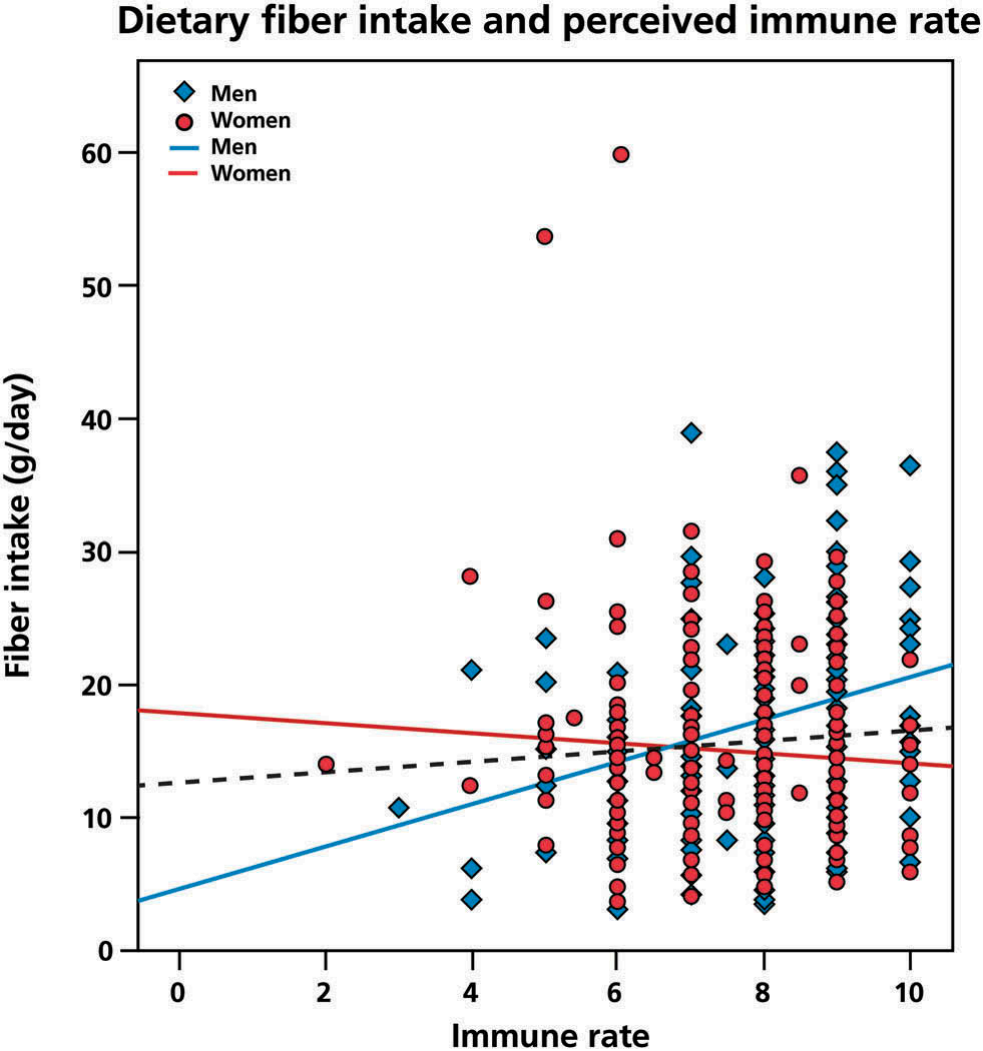
Dietary fiber intake and perceived immune functioning. To investigate the potential association between dietary fiber intake and perceived immune functioning, a two-tailed nonparametric Spearman’s correlation was used. Positive correlations were found for the total study population (*p *= 0.008) and for men (*p *= 0.002). There was no significant relationship between dietary fiber intake and perceived immune rate in women.

Figures 1 and 2 show the relationship between daily fiber intake and perceived health and immune status. Daily total fiber intake correlated significantly with the general health rate (r = 0.171, *p *= 0.0001) and perceived immune functioning (r = 0.124, *p *= 0.008). The partial correlation between fiber intake and general health remained significant (r = 0.151, *p *= 0.002) after controlling for total caloric intake.

In men, dietary fiber intake correlated significantly with perceived general health status (r = 0.320, *p *= 0.0001) and immune functioning (r = 0.281, *p *= 0.002). After controlling for caloric intake, the association between dietary fiber intake and perceived general health (r = 0.261, *p *= 0.005) remained significant, whereas the relationship with immune functioning (r = 0.165, *p *= 0.077) appears to be partly moderated by caloric intake. No significant relationship was found between dietary fiber intake and perceived health and immune rate in women.

Daily intake of cereals and grain products correlated significantly with perceived immune functioning (r = 0.110, *p *= 0.14), but not with general health ratings. Daily intake of vegetables or fruit did not correlate significantly with perceived immune functioning or general health ratings. The ratio between protein and carbohydrates correlated significantly with perceived immune functioning (r = − 0.121, *p *= 0.013) but not with health rate. No significant difference in protein–carbohydrate ratio was observed between men and women.

## Discussion

The findings from this study are consistent with previous reports in regards to fiber intake and the relationship between fiber intake and health and immune status.[9] Furthermore, it adds to the literature by showing profound gender differences in these associations. Whereas significant associations between fiber intake and perceived health and immune status were found among men, these were not significant among women. In a population consisting of healthy young adults, large effects on health parameters would be unexpected. As anticipated, the average health and immune rates were high for both sexes. Nevertheless, a significant gender difference was found in the associations between fiber intake and health and immune status.

The observed gender differences may be explained by differences in dietary habits. Previous studies have reported greater effects in men than in women.[19] This appears to be related to women having baseline dietary habits more concordant with principles of the dietary interventions compared to men.[19,20] In contrast, men with lower daily fiber intake may benefit from dietary changes towards fiber-rich food.

Consumption of fibers from cereals, fruits and vegetables show different health benefits,[21] including mental benefits.[22] The fact that women consumed significantly more fibers from fruit, whereas men consumed significantly more fibers from cereals and grains may explain the observed gender differences.

There is evidence for substantial individual variation in gut microbial composition. Interestingly, a gender difference in dietary intake on microbiota has been observed for various vertebrates, including humans, with a consistent trend for larger dietary effects in males than in females. Although the underlying mechanisms for gender modulation of diet effects remain uncertain, mammalian studies have shown that sex hormones are able to modulate the microbial composition.[23,24] Additionally, immune function and susceptibility to immune related disease differ between the genders.[25,26] Hence, sex hormones and gender differences in immune function could possibly be the underlying reasons to the microbial differentiations and the different dietary effects.[27] This is further supported by animal studies showing that it is possible to suppress autoimmunity in high genetic risk animals through alteration of the gut microbiome. Additionally, sex hormones appear to be able to modulate sexual dimorphism observed in human autoimmune disease.[23] Although the observed effects in our relatively healthy and young population were small, a gender-specific impact of diet on the gut microbiota may explain the sex differences in perceived immune functioning. Since evidence of intestinal dysbiosis in autoimmune disease is emerging and alterations of the microbiota appear to be a causal factor, nonpathogenic sex-specific microbial therapies could be a future therapeutic option in immune related disease.[28–31]

The mean daily dietary intake of fibers in our study population is in line with previous findings showing that Western diets contain fewer fibers than recommended. Besides dietary fiber intake, the association between self-reported health rate and fiber intake was also consistent with the NHANES results (15.5–16.1 g/day), as presented by King et al. [9]

The uneven ratio of men and women (1:3) in this study reflects the actual gender distribution of the Utrecht student population.[32] Furthermore, the cohorts were large enough to achieve sufficient power to be confident about the study outcome despite the gender ratio.

Although it is important to have objective assessments of immune functioning, people are often judging their health based on *perceived* health status. By tradition, health has been measured in mortality, morbidity, incidence and prevalence of disease, and so on. However, physical, emotional and social conditions all have a role in the etiology of disease and health perception should therefore not be ignored. Health decisions may be in part dependent on perceived health status.[33,34] In this regard, we agree with the World Health Organization who define health as a subjective state or ‘A complete state of physical, mental and social well-being, and not merely the absence of disease or infirmity.’[35] Nevertheless, the lack of laboratory assessments and physical examinations presents certain limitations to this study. Objective assessment should therefore be conducted in future studies.

Although the FFQ provides a measure of dietary fiber intake, it remains an estimate of true consumption. For example, the current FFQ did not account for portion sizes. Future studies should take this into account. Nevertheless, similar estimates have been used successfully in previously studies with young adults investigating the impact of food on perceived stress and depressive symptoms.[13–15] A general concern in dietary studies using self-report is the issue of deliberate or unconscious under-reporting.[36,37] In this study, a considerable low caloric intake was observed for both men and women. This may be explained by an underestimation in fat measurements by the FFQ. Also, to improve the reporting accuracy, energy intake cutoff values can be used to exclude under- or over-reporting subjects. While these values have been used in earlier settings, energy expenditure was not taken into account in the current study. This could have an effect on the validity of the dietary assessment.[18] In future research it will be important to take this into account, for example by applying the Goldberg cutoff that uses the ratio of energy intake to estimated basal metabolic rate to filter out unreliable reporting.[13,36] Nevertheless, since total energy intake may confound associations between specific nutrients and disease risk, bivariate analyses were applied in order to correct for caloric intake.[38] Finally, in the current study we did not take consumption of fiber supplements into consideration. Nonetheless, earlier studies have demonstrated that for the vast majority of people fiber supplements contribute very little to the total intake of dietary fibers.[9,39]

Taken together, the findings from this study suggest that there is a modest association between dietary intake of fibers and perceived general health and immune functioning in men but not in women. Prospects for microbiome manipulation to treat diseases that originate from dysbiosis are of growing interest.[21,28] The use of environmental factors such as diets may lead to therapeutic alteration of the microbiome in a cost-effective manner.[27] Taking into account the limitations of the current study, future research is needed to further investigate the possible effects of gender differences. Combining subjective measurements with laboratory assessments including objective analyses of microbial composition and immune parameters are necessary to further explore the impact of fiber intake on health and immune status.
